# Prevalence of Colorectal Cancer Screening Among Latino Adults Following the Medicaid Eligibility Amendment Expansion

**DOI:** 10.1001/jamanetworkopen.2025.59100

**Published:** 2026-02-11

**Authors:** Nathalie Huguet, Jorge Kaufmann, Heather Holderness, Jeremy Erroba, Gretchen Mertes, Teresa Schmidt, Miguel Marino, John Heintzman

**Affiliations:** 1 Department of Family Medicine, Oregon Health and Science University, Portland; 2 Department of Innovation and Improvement, OCHIN, Portland, Oregon

## Abstract

**Importance:**

Cancer is the leading cause of death for Latino and Latina individuals in the US. Lack of insurance is one of the leading contributing factors to low prevalence of colorectal cancer screening. In 2021, the state Medicaid coverage programs in California and Oregon were amended to expand income-based eligibility for Medicaid to adults aged 50 years or older regardless of immigration status.

**Objective:**

To evaluate whether expanding Medicaid eligibility regardless of immigration status to adults aged 50 years or older with low income was associated with improvements in the prevalence of up-to-date colorectal cancer screening among Latino and Latina patients who received care at community health centers.

**Design, Setting, and Participants:**

This retrospective case-control study used electronic health record data from clinics from January 2018 to December 2023 in states that expanded Medicaid eligibility regardless of immigration status by 2021 (California and Oregon) and from those that did not amend their eligibility (Indiana, Minnesota, Ohio, and Washington). The dataset included Latino and Latina patients aged 50 to 63 years who were uninsured at baseline and had visits both before (2018-2019) and after (2021-2023) the Medicaid eligibility amendment (excluding 2020 because of the COVID-19 pandemic and its association with primary care delivery).

**Exposure:**

State adoption of the 2021 Medicaid income-based eligibility amendment regardless of immigration status to adults aged 50 years or older.

**Main Outcomes and Measures:**

The prevalence of up-to-date colorectal cancer screening was assessed using a doubly robust covariate-adjusted difference-in-differences model to estimate the average treatment effect on the treated (ATT).

**Results:**

There were 6503 Latino and Latina patients (mean [SD] age, 54.04 [3.32] years; 3623 females [55.7%]) from 218 clinics included in the study, of whom 5957 (91.6%) preferred the Spanish language. The prevalence (in percentage points) of colorectal cancer screening increased among uninsured English-preferring Latino and Latina patients (ATT = 19.53 [95% CI, 9.04-30.02]), English-preferring Latino men (ATT = 16.72 [95% CI, 1.27-32.16]) when considering all 3 years of the posteligibility period, and Spanish-preferring Latina women (ATT = 8.58 [95% CI, 1.48-15.67]) in the third posteligibility year in eligibility amendment states more than their counterparts in nonamendment states.

**Conclusions and Relevance:**

In this case-control study of Latino and Latina patients, expansion of Medicaid eligibility was associated with significantly higher prevalence of colorectal cancer screening rates among Spanish-preferring Latina women and English-preferring Latino and Latina patients. The findings suggest that state-funded coverage expansions may help increase colorectal cancer–screening prevalences in this population.

## Key Points

**Question:**

Was expanding Medicaid eligibility to all adults regardless of immigration status associated with an improvement in the prevalence of colorectal cancer screening among Latino and Latina adults?

**Findings:**

In this case-control study of 6503 Latino and Latina patients, expanding Medicaid regardless of immigration status was associated with increased prevalence of colorectal cancer screening among uninsured Latina patients with a Spanish-language preference and Latino and Latina patients with an English-language preference.

**Meaning:**

The findings of this study suggest that state-funded insurance coverage expansions may help increase colorectal cancer–screening prevalences among Latino and Latina adults.

## Teaser

This case-control study examines whether there were changes in the prevalence of colorectal cancer screening among Latino and Latina adults following amendments in California and Oregon that expanded Medicaid eligibility to adults aged 50 years and older regardless of immigration status.

## Introduction

Cancer is the leading cause of death for Latino and Latina individuals in the US,^1^ who represent approximately 19% of the entire US population.^2^ Specifically, colorectal cancer is the second-most common cancer and cause of cancer deaths among Latino men^3^ and the third-most common cancer and cause of cancer deaths among Latina women.^4^ Despite advances in technology for early detection and screening, early onset of colorectal cancer, before the age of 50 years, disproportionately burdens Latino and Latina individuals at a rate nearly double that of non-Hispanic White individuals.^5^ Latino and Latina individuals are less likely to be up to date with colorectal cancer screening compared with White individuals (prevalence: 52% vs 61%, respectively), especially for individuals who are uninsured compared with those who are insured (15% vs 24%).^4^ There are many barriers to colorectal cancer screening, and the lack of insurance coverage is one of the largest contributing factors.^3,6,7^

Federal and state policy initiatives in the last decade, such as the Patient Protection and Affordable Care Act (ACA) and other state Medicaid expansion programs, have expanded coverage to populations with socioeconomic disadvantage. The ACA substantially increased access to health insurance and mandated the coverage of preventive services with no out-of-pocket cost for patients. These policies have been shown to lead to a greater uptake of health insurance as well as increases in access to preventive care and better health outcomes among people living in or near poverty.^8,9,10,11,12,13^ However, Latino and Latina individuals, along with those who are American Indian or Alaska Native, continue to be more likely to be uninsured than other groups in the US.^14,15,16,17^ It was estimated in 2022 that 30% of Latino and Latina immigrants are undocumented, of whom half were likely uninsured because they are not eligible for any public-funded insurance or Marketplace insurance.^18,19,20^ One exception is emergency Medicaid, which is temporary. Additionally, even though some Latino and Latina individuals are eligible for health insurance programs (such as Medicaid), they remain uninsured because of the multiple immigration policy changes, which have led to fear^21,22,23,24^ and misunderstanding among Latino and Latina communities with mixed immigration status.^25^

Over the past few years, several states (eg, California, Colorado, Oregon, and Washington) have amended their health insurance program eligibility (public or Marketplace) to include all adults with low income regardless of immigration status. Specifically, in 2021, California and Oregon amended their state Medicaid coverage programs to expand income-based eligibility for Medicaid to all adults aged 50 years or older who were income eligible regardless of immigration status^26,27^; as of 2024, California and Oregon have expanded to all adults. A recent study showed increases in Medicaid-covered visits among Latino and Latina patients following these eligibility amendments in California and Oregon^28^; these improvements in insurance access may facilitate uptake of colorectal cancer screening. Additionally, data published in Oregon show an uptake in Medicaid enrollment for more than 100 000 immigrants.^29^ However, changes to Medicaid, the ACA, and health insurance exchange programs (ie, Marketplace), as a result of the reconciliation bill, which became law in July 2025,^30^ could substantially impede or even reverse progress made in extending equitable access to care for the Latino communities.^2^ Evaluating the association of these eligibility amendments with up-to-date cancer screening is important to inform policymakers.

This study assesses whether expanding Medicaid eligibility to adults aged 50 years or older with low income, regardless of their immigration status, was associated with higher prevalence of up-to-date colorectal cancer screening among Latino and Latina patients who received care at community health centers. Community health centers provide care to 31.5 million patients, wherein 1 in 6 patients is a Medicaid beneficiary, 1 in 3 lives in poverty, and more than 1 in 3 are Latino or Latina individuals.^31^ We compared changes in the prevalence of up-to-date colorectal cancer screening before and after eligibility amendments in California and Oregon relative to states without the eligibility amendment (but with the ACA Medicaid expansion). We compared these changes among Latino and Latina patients by language preference (Spanish or English) and sex (male or female). We chose to disaggregate by language preference because preferred language has been shown to be a proxy for acculturation and associated with differing health care utilization.^28,32,33,34,35,36,37^ In addition, evidence shows that Latino men are less likely to seek care than are Latina women; therefore, we evaluated changes in Medicaid coverage separately for men and women.^38,39^ We hypothesized that Latino and Latina individuals in eligibility-amendment states would be associated with larger improvements from before to after the eligibility amendment in the prevalence of up-to-date screening relative to those in nonamendment states.

## Methods

This retrospective case-control study centered on California and Oregon, which expanded their Medicaid eligibility in 2021 to all adults aged 50 years or older regardless of immigration status. As a comparison, we included states that did not amend their Medicaid eligibility but had expanded Medicaid to adults with low income in accordance with the ACA Medicaid expansion by the start of the study period, had clinics with at least 20 Latino and 20 Latina patients on their panel, did not have other insurance programs (eg, local program or Marketplace) supporting people ineligible for Medicaid or Marketplace insurance, and participated in the OCHIN network in the period before and after the expansion. These states included Indiana, Minnesota, Ohio, and Washington. The Oregon Health and Science University institutional review board approved the study and waived informed consent because of use of deidentified data. This study followed the Strengthening the Reporting of Observational Studies in Epidemiology (STROBE) reporting guideline.

### Data Source

Patient-level data were from the OCHIN Epic electronic health record (EHR) system. OCHIN provides a fully hosted and shared EHR platform across a nationwide network of health centers. OCHIN is part of the Accelerating Data Value Across a National Community Health Center Network (ADVANCE) clinical research network of The National Patient-Centered Clinical Research Network (PCORnet).^40^ ADVANCE clinical data are routinely assessed for completeness and quality following PCORnet’s standard analytic queries and data quality-check process and have low missingness on relevant variables. All study data, including patient self-reported ethnicity and preferred language, were collected in the routine course of clinical care in structured, preexisting fields in the OCHIN Epic EHR system. We used the labels Latino and Latina because they are often preferred in our study population; however, the actual ethnicity information collected by the clinics is most often categorized as Hispanic and non-Hispanic.

### Population

The study population consisted of Latino and Latina adults who had an uninsured visit in the period before the Medicaid eligibility expansion and were aged 50 to 63 years during the study period from January 2018 to December 2023 to align with the eligibility criteria of the amendment in California and Oregon. Notably, in 2021 the US Preventive Services Task Force adjusted the recommended age for screening earlier to age 45 years. We studied adults aged 50 years or older to align with the change in the Medicaid policy in an age group still targeted for screening. Participants received primary care within the OCHIN network, defined as having visits with a physician (allopath, osteopath, or naturopath), physician assistant, or nurse practitioner and billed with any of the following *Current Procedural Terminology* codes for a primary care visit: 99201-99205, 99212-99215, 99241-99245, 99386-99387, and 99396-99397. We included patients who were established in 2018 and active at least 1 year in the postamendment period (2021-2023). A patient was considered established in 2018 if they had a primary care visit within a 3-year period^41^ of 2018 (ie, 2016-2018, 2017-2019, or 2018-2020). Patients were excluded if they were pregnant during the study (n = 8) because pregnant individuals have different eligibility criteria for Medicaid than other adults. Patients with a colorectal cancer diagnosis or colectomy prior to the study start (n = 48) were also excluded. Patients were excluded if they had missing data on language and sex.

### Dependent Variables

The outcome measure was defined as the prevalence of up-to-date colorectal cancer screening for each calendar year of the study. Patients were considered up to date on screening if they met any of the following criteria: a fecal immunochemical test (FIT) or a guaiac fecal occult blood test (FOBT) in the current year of measurement, a flexible sigmoidoscopy in the current year or in any of the prior 4 calendar years with up-to-date colorectal cancer screening, or a colonoscopy in the current year or in any of the prior 9 calendar years. Of note, a computed tomography colonography was not considered for up-to-date status because there were no records of the procedure among our sample. These criteria for colorectal cancer–screening status were determined using EHR documentation of diagnostic codes, lab LOINC (Logical Observation Identifiers Names and Codes), and *Current Procedural Terminology* codes (listed in eTable 1 in Supplement 1).

### Independent Variables

The primary exposure variable was a binary variable (amendment group), indicating whether a patient’s residential location was in a state that amended income-based eligibility to adults aged 50 years or older regardless of immigration status (California and Oregon) or in a state that did not amend eligibility (Indiana, Minnesota, Ohio, and Washington). The main independent variables differentiated a patient’s sex and preferred language (Spanish or English).

### Covariates

Sociodemographic information derived from the EHR and used as covariates in adjusted regression-based analyses included age at first study visit; last known household income as a percentage of the federal poverty level; experiencing homelessness during the study; number of primary care visits per year; baseline insurance status in the preamendment period; whether the patient had a diagnosis of irritable bowel syndrome, inflammatory bowel disease, or family history of colorectal cancer; and the presence of chronic conditions on a patient problem list. The chronic conditions considered included alcohol use disorder, tobacco use, drug use disorder, asthma, chronic obstructive pulmonary disorder, diabetes, mental health disorder (anxiety, depression), and obesity.^42^

### Statistical Analysis

First, we described the characteristics of the sample and subgroups defined by preferred language. We estimated and produced profile plots depicting unadjusted language-stratified prevalence of up-to-date colorectal cancer screening for each year over the study period overall and stratified by sex.

Additionally, for all Latino and Latina individuals, we plotted the disaggregated prevalence of the screening method (FIT or FOBT or colonoscopy) utilized by year. Next, we used the doubly robust covariate-adjusted difference-in-differences approach proposed by Sant’Anna and Zhao^43^ to evaluate the association of eligibility amendment with the prevalence of up-to-date colorectal cancer screening. This approach combined outcome regression with propensity score weighting to address potential model misspecification, heterogeneity of treatment effects across patients, and efficiency concerns.^43^ Specifically, outcome regression utilized a standard difference-in-differences ordinary least-squares estimator with an indicator for amendment group (states that expanded eligibility regardless of immigration status vs states that did not expand eligibility), study year (before vs after), and their interaction. Analyses were conducted at the patient level for binary outcomes. Ordinary least-squares estimators are often preferred for binary outcomes for causal inference approaches, as is the case in this study. This outcome regression was weighted with inverse probability scores derived from logistic regression of the amendment group on preperiod covariates. SEs accounted for clustering of multiple observations within patients. We report the average treatment effect on the treated (ATT), linear combinations of dynamic treatment effects separately for each year following the start of the Medicaid eligibility amendment (2021, 2022, and 2023), and corresponding 95% CIs for both English language-preferring and Spanish language-preferring Latino and Latina individuals overall and stratified by sex.

Data from 2020 were depicted in the plot profiles but excluded from the difference-in-differences analysis due to the COVID-19 pandemic and its association with primary care delivery. The COVID-19 pandemic year 2020 had different visit trends with significant reductions in March to June in overall care and cancer prevention across US states, followed by a recovery period to near-baseline level by October 2020.^44,45^ Both amendment and nonamendment states experienced these trends. Parallel trends between the eligibility amendment groups were visually inspected using adjusted event study plots and interaction tests using preperiod data. In our evaluation of the parallel trends assumption (eTable 2 and eFigure 1 in Supplement 1), we found that the parallel trends assumption holds for all comparisons.

In addition, while our primary analysis included patients and clinics that were not exposed to the 2021 eligibility amendment as a comparison group, it is possible to conceptualize an alternative comparison group. Specifically, we could compare Latino and Latina patients with non-Hispanic White patients within California and Oregon. This comparison introduces external confounding that the data cannot adjust for regarding barriers to colorectal cancer screening such as cultural belief, trust, and structural challenges (eg, immigration policies). We performed this sensitivity analysis by replacing our original comparison group with non-Hispanic White patients within California and Oregon. We then redid our difference-in-differences approach replacing our primary exposure with ethnicity group (Latino and Latina vs non-Hispanic White individuals who preferred the English language).

The analytic dataset was created using R, version 4.3.0 (R Project for Statistical Computing). Analyses were conducted with Stata, version 18.0 (StataCorp LLC) using a 2-sided *P* < .05 statistical significance threshold and 2-sided testing with a type I error (α = .05).

## Results

Among the 6503 Latino and Latina patients (mean [SD] age, 54.04 [3.32] years; 3623 females [55.7%] and 2880 males [44.3%]) from 218 clinics in the sample, 5957 (91.6%) preferred the Spanish language, and 546 (8.4%) preferred the English language. The sample had a higher proportion of Latina women than Latino men. Most patients were at or below 138% of the federal poverty level (4311 [66.3%]), and most received care in an eligibility amendment state (5661 [87.1%]) (Table 1).

**Table 1.** Characteristics of Latina and Latino Individuals, Who Were Uninsured in the Period Before the Medicaid Eligibility Amendment Receiving Care in Community-Based Clinics, by Eligibility Amendment Status, From 2018 to 2023^a^

Patient characteristics	Patients, No. (%)		
	Total (N = 6503)	States without eligibility amendment (n = 842)	States that amended eligibility (n = 5661)
Sex			
Female	3623 (55.7)	472 (56.1)	3151 (55.7)
Male	2880 (44.3)	370 (43.9)	2510 (44.3)
Age at baseline, y			
50-54	3820 (58.7)	515 (61.2)	3305 (58.4)
55-63	2683 (41.3)	327 (38.8)	2356 (41.6)
Last known income, % FPL			
≤138	4311 (66.3)	638 (75.8)	3673 (64.9)
>138	1568 (24.1)	178 (21.1)	1390 (24.6)
No information	624 (9.6)	26 (3.1)	598 (10.6)
Experiencing homelessness ever during study	436 (6.7)	50 (5.9)	386 (6.8)
No. of clinic visits per y			
<2	3355 (51.6)	471 (55.9)	2884 (50.9)
2-3	2109 (32.4)	269 (31.9)	1840 (32.5)
≥4	1039 (16.0)	102 (12.1)	937 (16.6)
Family history of colorectal cancer	99 (1.5)	21 (2.5)	78 (1.4)
IBS or IBD diagnosis	281 (4.3)	27 (3.2)	254 (4.5)
No. of chronic conditions			
0	2070 (31.8)	324 (38.5)	1746 (30.8)
1	2485 (38.2)	298 (35.4)	2187 (38.6)
≥2	1948 (30.0)	220 (26.1)	1728 (30.5)
Up-to-date colorectal cancer screening in 2019	2281 (35.1)	366 (43.5)	1915 (33.8)
Language preference			
Spanish	5957 (91.6)	715 (84.9)	5242 (92.6)
English	546 (8.4)	127 (15.1)	419 (7.4)

Abbreviations: FPL, federal poverty level; IBD, inflammatory bowel disease; IBS, irritable bowel syndrome.

^a^ Data from 218 clinics in amendment states (California and Oregon) and in nonamendment states (Indiana, Minnesota, Ohio, and Washington) were presented before the Medicaid eligibility amendment (2018-2019) and after the Medicaid eligibility amendment (2021-2023) among persons aged 50 to 63 years.

Overall, among the entire study sample over the course of 2018 to 2023, the unadjusted prevalences for being up to date on colorectal cancer screening were lowest in 2020 (Latina women: 29.1%; Latino men: 24.0%) and highest in 2023 (Latina women: 39.4%; Latino men: 35.1%) (eFigure 2 in Supplement 1). Among both Latina and Latino individuals, a FIT or a FOBT accounted for most of the screenings prior to 2020. However, the percentage receiving a colonoscopy increased in each year of the study and accounted for most of the screenings after 2020 (eFigure 2 in Supplement 1).

**Figure 1.**
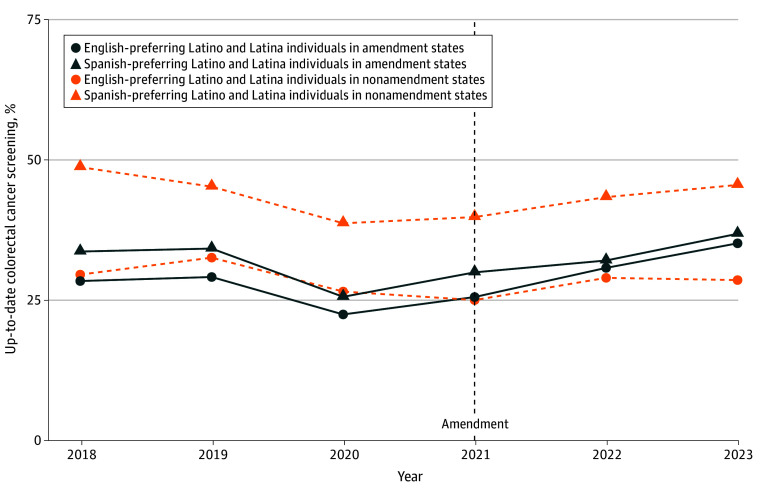
Yearly Unadjusted Prevalence of Up-to-Date Colorectal Cancer Screening Among English Language-Preferring and Spanish Language-Preferring Latino and Latina Individuals in Amendment and Nonamendment States From 2018 to 2023 Data from 218 clinics in amendment states (California and Oregon) and in nonamendment states (Indiana, Minnesota, Ohio, and Washington) were presented before the Medicaid eligibility amendment (2018-2019) and after the Medicaid eligibility amendment (2021-2023) among persons aged 50 to 63 years.

**Figure 2.**
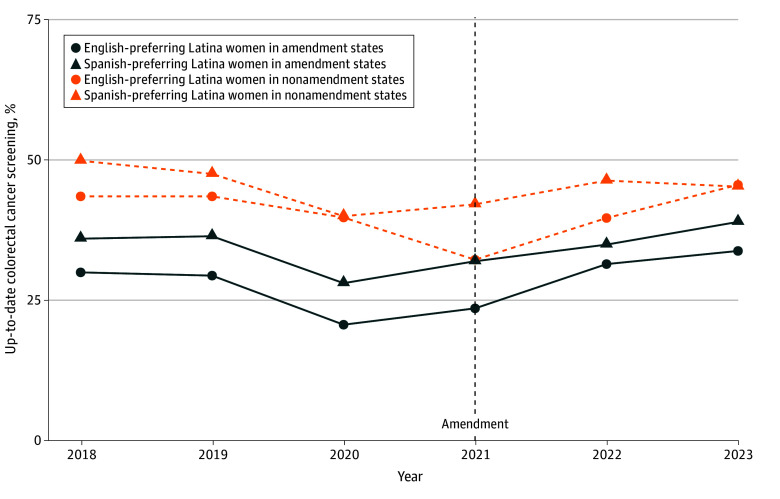
Yearly Unadjusted Prevalence of Up-to-Date Colorectal Cancer Screening Among English Language-Preferring and Spanish Language-Preferring Latina Women in Amendment and Nonamendment States From 2018 to 2023 Data from 218 clinics in amendment states (California and Oregon) and in nonamendment states (Indiana, Minnesota, Ohio, and Washington) were presented before the Medicaid eligibility amendment (2018-2019) and after the Medicaid eligibility amendment (2021-2023) among persons aged 50 to 63 years.

**Figure 3.**
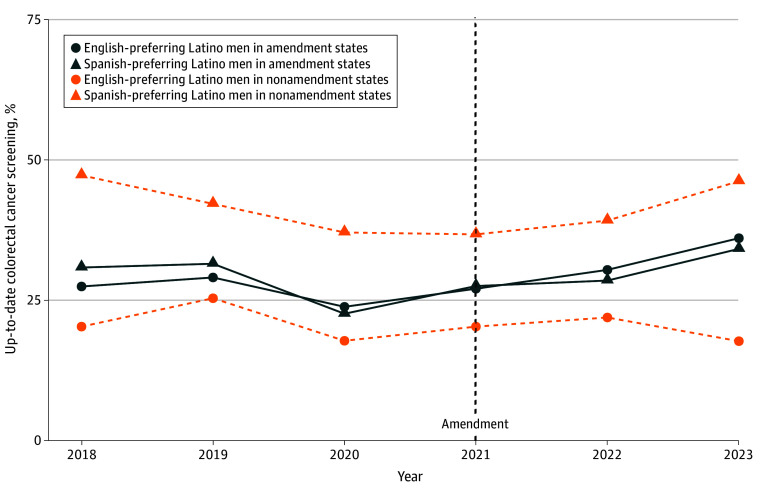
Yearly Unadjusted Prevalence of Up-to-Date Colorectal Cancer Screening Among English Language-Preferring and Spanish Language-Preferring Latino Men in Amendment and Nonamendment States From 2018 to 2023 Data from 218 clinics in amendment states (California and Oregon) and in nonamendment states (Indiana, Minnesota, Ohio, and Washington) were presented before the Medicaid eligibility amendment (2018-2019) and after the Medicaid eligibility amendment (2021-2023) among persons aged 50 to 63 years.

The yearly unadjusted prevalences for up-to-date colorectal cancer screening overall and stratified by sex among those uninsured in the period before Medicaid expansion are shown in Figure 1, Figure 2, and Figure 3. All groups in amendment and nonamendment states experienced a drop in prevalence in 2020, and prevalences were below 50%. A steady increase of the prevalences of being up to date with colorectal cancer screening in 2021 and 2022 for both Spanish-preferring and English-preferring Latino and Latina individuals is shown in Figure 1. In states that did not amend eligibility, prevalences of being up to date with colorectal cancer screening increased in 2022 and 2023 among Spanish-preferring Latino and Latina individuals and showed small changes among English-preferring Latino and Latina individuals together (from 25.0% in 2021 to 28.6% in 2023).

Among Latina women, prevalences of being up to date steadily increased in 2022 and 2023 for those who both preferred Spanish and English languages in eligibility amendment states (Figure 2). In states that did not amend eligibility, prevalences of being up to date with colorectal cancer screening increased in 2022, but prevalence only continued to increase in 2023 for the English-preferring Latina women who were uninsured at baseline.

Latino men’s unadjusted up-to-date prevalences of colorectal cancer screening and increases in prevalences among those who both preferred Spanish and English languages in eligibility amendment states are shown in Figure 3. In nonamendment states, English-preferring Latino men had much lower prevalence of up-to-date colorectal cancer screening than Spanish-preferring Latino men over the period, and both groups showed small increases in 2022 and either an increase (Spanish preferring) or a small decline (English preferring) in 2023.

Results from the difference-in-differences models are presented in Table 2. In the model combining males and females, English-preferring Latino and Latina individuals in eligibility amendment states had a significantly greater increase in prevalences of being up to date with colorectal cancer screening than did English-preferring Latina women in nonamendment states (ATT = 19.53 [95% CI, 9.04-30.02]), when aggregating across the full postamendment period. That is, the prevalence of up-to-date status increased 19.53 percentage points more for those in amendment states compared with those in nonamendment states. This pattern of results was also observed among English-preferring Latino men when aggregating across the full postamendment period (ATT = 16.72 [95% CI, 1.27-32.16]) and among Spanish-preferring Latina women in the third year after the amendment (8.58 [95% CI, 1.48-15.67]). There were no significant changes in prevalence of being up to date with colorectal cancer screening among Spanish-preferring Latino and Latina individuals, English-preferring Latina women, and Spanish-preferring Latino men in comparing amendment with nonamendment states.

**Table 2.** ATT of the 2021 Medicaid Eligibility Amendment on the Prevalence of Being Up to Date for Colorectal Cancer Screening Among Latino and Latina Patients, Who Were Uninsured in the Period Before the Amendment, by Language Preference and Sex^a^

Group	ATT, posteligibility amendment period, percentage points (95% CI)			
	First y (2021)	Second y (2022)	Third y (2023)	All 3 y combined
**Latina and Latino patients**				
Spanish-preferring	−0.58 (−5.14 to 3.98)	0.85 (−4.09 to 5.80)	2.44 (−3.25 to 8.14)	0.91 (−3.41 to 5.22)
English-preferring	10.94 (1.11 to 20.77)	17.53 (5.85 to 29.22)	30.12 (14.72 to 45.51)	19.53 (9.04 to 30.02)
**Latina women**				
Spanish-preferring	2.07 (−3.65 to 7.79)	2.97 (−4.20 to 10.15)	8.58 (1.48 to 15.67)	4.54 (−0.91 to 9.99)
English-preferring	1.58 (−10.49 to 13.64)	−1.60 (−20.19 to 16.98)	−2.51 (−26.17 to 21.15)	−0.85 (−16.18 to 14.49)
**Latino men**				
Spanish-preferring	−1.09 (−7.75 to 5.57)	2.55 (−4.18 to 9.27)	−0.27 (−8.37 to 7.82)	0.39 (−5.66 to 6.45)
English-preferring	6.92 (−7.61 to 21.45)	17.98 (2.73 to 33.23)	25.26 (3.11 to 47.41)	16.72 (1.27 to 32.16)

Abbreviation: ATT, average treatment effect on the treated.

^a^ Models were adjusted for age, household income, experiencing homelessness, clinic visits per year, family history of colorectal cancer, irritable bowel syndrome or inflammatory bowel disease diagnosis, chronic conditions, and baseline insurance status. Prevalences were evaluated as the number of patients up to date on screening per 100 patients, and the reported ATT values reflect differences in percentage-point change of rates of being up to date. Data from 218 clinics in amendment states (California and Oregon) and in nonamendment states (Indiana, Minnesota, Ohio, and Washington) were presented before the Medicaid eligibility amendment (2018-2019) and after the Medicaid eligibility amendment (2021-2023) among persons aged 50 to 63 years.

The sensitivity analysis that compared 2364 English-preferring non-Hispanic White individuals with Latino and Latina individuals within California and Oregon showed a higher baseline prevalence of up-to-date colorectal cancer screening among Spanish-preferring and English-preferring Latino and Latina individuals, although all were below 50% (eTable 3 and eFigure 3 in Supplement 1). Spanish-preferring Latino and Latina individuals experienced the largest decline in 2020. All groups showed steady increases from 2021 to 2023. Difference-in-differences analyses that included non-Hispanic White individuals within amendment states as the controls showed significantly greater increases in prevalences of being up to date with colorectal cancer screening among non-Hispanic White individuals relative to Latino and Latina individuals regardless of language preference (ATT = −5.27 [95% CI, −7.94 to −2.61]) and relative to Spanish-preferring Latino and Latina individuals (ATT = −5.31 [95% CI, −8.04 to −2.59]). There were no significant changes in prevalence of being up to date with colorectal cancer screening when comparing English-preferring Latino and Latina individuals with English-preferring non-Hispanic White individuals.

## Discussion

This case-control study evaluated whether expansion of the Medicaid eligibility amendment was associated with improvements in the prevalence of being up to date with colorectal cancer screening among Latino and Latina patients aged 50 to 63 years. The study found a modest increase in the prevalence of being up to date with colorectal cancer screening among uninsured Latino and Latina individuals who prefer English in amendment states compared with their counterparts in nonamendment states. These results align with previous studies focusing on the ACA Medicaid expansion, which showed increases in colorectal cancer–screening rates after reforms.^10,33^ These favorable outcomes are important for a population in which cancer is the leading cause of death. However, current immigration policies and upcoming changes to health insurance eligibility (public, exchange) threaten these expansions. In May 2025, the California governor proposed freezing enrollment and imposed a $100 monthly premium for those currently enrolled in the full-scope expansion.^46^ Additionally, the Centers for Medicare & Medicaid Services has been ordered by the Trump administration to provide identifiable data to homeland security or face funding consequences.^47^ Threats such as these may lower access to care among the Latino and Latina community in general and may decrease cancer prevention and worsen colorectal cancer outcomes in the coming years.

Although the findings showed differences in the prevalence of up-to-date colorectal cancer screening when comparing Latino and Latina individuals in amendment vs nonamendment states, the prevalence of being up to date with colorectal cancer screening among English-preferring non-Hispanic White individuals compared with Latino and Latina individuals was associated with greater improvements within amendment states. These findings suggest that although the amendment may have been associated with greater improvement in the prevalence of up-to-date colorectal cancer screening within the Latino and Latina population, it did not exceed the prevalence rise among non-Hispanic White individuals.

The COVID-19 pandemic was associated with a larger drop in colorectal cancer screening among Latino and Latina individuals than among non-Hispanic White individuals, as seen in eFigure 3 in Supplement 1. During the pandemic, there was a large increase in use of mailed FIT, which may explain increases in colorectal cancer screening among non-Hispanic White individuals.^44^ However, this heightened use of mailed FIT during the pandemic occurred in both amendment and nonamendment states^44^ and would not explain the differential prevalence within the Latino and Latina population by amendment status.

Our findings showed differential outcomes by sex. Uninsured Latino men had a particularly low prevalence of colorectal cancer–screening completion, but screening was associated with improvements among English-preferring Latino men. In contrast, increased prevalences were seen among Spanish-preferring Latina women. Previous studies have highlighted that Latina women are more likely to seek care and have health insurance, often associated with reproductive care, whereas Latino men’s health care decisions may be influenced by a perception of illness and gender expectations and norms (which have been shown to be associated with reduced care or preventive-service seeking), which could have driven these differences.^38,39,48,49^ There is evidence that cultural barriers and stigma are substantial impediments for Latino men, and these results suggest that gaining health insurance alone is insufficient in producing notable changes among Spanish-preferring Latino men.^3^ A previous study noted the importance of peer and family in influencing uptake in screening.^3^ Strategies to increase colorectal cancer screening among Latino men may necessitate developing clinic protocols for them that involve peer and family testimonials on the benefits of screening and leverage multigenerational household dynamics to support screening behaviors.^50^ Additional research is needed to develop and evaluate such strategies.

In addition, although the prevalences of up-to-date colorectal cancer screening were relatively low in all groups (<50%), they were greater in nonamendment states across the years, especially for Spanish-preferring Latino and Latina individuals. Within these states, clinics or public health agencies may have had different initiatives to improve cancer screening that targeted Latino communities. For example, Marion County, Indiana, offers free FIT kits to Latino community members who can pick up the test regardless of insurance status.^51^

### Limitations

This study has limitations. Data analyzed were from people who have received care in the OCHIN network clinics; data on those who enrolled in Medicaid but did not seek care or received care elsewhere were not available. The sample size is constrained to those aged 50 to 63 years, which limits statistical power and may underestimate the associations. Analyses of data in more recent years (2024-2025) would have allowed inclusion of additional states that have since expanded their eligibility (Washington and Colorado), and more research on younger age groups is needed, given the high colorectal cancer risk among Latino and Latina individuals younger than 50 years.^5^ Although alternative study designs (eg, interrupted time-series methods) could have been considered, COVID-19 pandemic–related disruptions limited reliable counterfactual trend estimation, making a difference-in-differences approach with comparison states more appropriate.

## Conclusions

The findings of this case-control study suggest that expanding Medicaid to uninsured low-income adults aged 50 years or older regardless of immigration status was associated with a modest increase in the prevalence of being up to date with colorectal cancer screening among English-preferring Latino and Latina individuals and Spanish-preferring Latina women. Spanish-preferring Latino men did not show these gains; additional intervention coupled with insurance reform may be necessary to raise the screening prevalence in this population. Upcoming changes to Medicaid and current immigration-related policies could undo the progress seen in California and Oregon and could lead to lower cancer screening across Latino and Latina communities.

## Notes

Supplement 1. **eTable 1. **Diagnostic, Lab LOINC, and *Current Procedural Terminology* Codes **eFigure 1.** Event Study Plots to Test for Preperiod Parallel Trends **eTable 2.** Statistical Testing of Preperiod Parallel Trends Assumptions **eFigure 2.** Unadjusted Percentage of Latino and Latina Individuals Up to Date on Colorectal Cancer Screening by Year and Sex Within the OCHIN Community Health Center Network **eTable 3.** Characteristics of English-Preferring Non-Hispanic White Individuals and English-Preferring and Spanish-Preferring Latino and Latina Individuals in California and Oregon Receiving Care in Community Health Clinics, 2018 to 2023 **eFigure 3.** Yearly Unadjusted Prevalence of Up-to-Date Colorectal Cancer Screening Among English-Preferring Non-Hispanic White and English-Preferring and Spanish-Preferring Latino and Latina Individuals, 2018 to 2023, Among Patients Uninsured at Baseline

Supplement 2.
Data Sharing Statement
