# Supplementary material for: Prevalence of Colorectal Cancer Screening Among Latino Adults Following the Medicaid Eligibility Amendment Expansion
Source: JAMA Netw Open. 2026 Feb 11;9(2):e2559100. doi: 10.1001/jamanetworkopen.2025.59100 (PMC12895285; doi:10.1001/jamanetworkopen.2025.59100)
Supplement: Supplement 1. — eTable 1. Diagnostic, Lab LOINC, and Current Procedural Terminology Codes eFigure 1. Event Study Plots to Test for Preperiod Parallel Trends eTable 2. Statistical Testing of Preperiod Parallel Trends Assumptions eFigure 2. Unadjusted Percentage of Latino and Latina Individuals Up to Date on Colorectal Cancer Screening by Year and Sex Within the OCHIN Community Health Center Network eTable 3. Characteristics of English-Preferring Non-Hispanic White Individuals and English-Preferring and Spanish-Preferring Latino and Latina Individuals in California and Oregon Receiving Care in Community Health Clinics, 2018 to 2023 eFigure 3. Yearly Unadjusted Prevalence of Up-to-Date Colorectal Cancer Screening Among English-Preferring Non-Hispanic White and English-Preferring and Spanish-Preferring Latino and Latina Individuals, 2018 to 2023, Among Patients Uninsured at Baseline [file jamanetwopen-e2559100-s001.pdf]

Supplemental Online Content

Huguet N, Kaufmann J, Holderness H, et al. Prevalence of colorectal cancer screening among Latino adults following the Medicaid eligibility amendment expansion. *JAMA Netw Open*. 2026;9(2):e2559100. doi:10.1001/jamanetworkopen.2025.59100

eTable 1. Diagnostic, Lab LOINC, and *Current Procedural Terminology* Codes
eFigure 1. Event Study Plots to Test for Preperiod Parallel Trends
eTable 2. Statistical Testing of Preperiod Parallel Trends Assumptions
eFigure 2. Unadjusted Percentage of Latino and Latina Individuals Up to Date on Colorectal Cancer Screening by Year and Sex Within the OCHIN Community Health Center Network
eTable 3. Characteristics of English-Preferring Non-Hispanic White Individuals and English-Preferring and Spanish-Preferring Latino and Latina Individuals in California and Oregon Receiving Care in Community Health Clinics, 2018 to 2023
eFigure 3. Yearly Unadjusted Prevalence of Up-to-Date Colorectal Cancer Screening Among English-Preferring Non-Hispanic White and English-Preferring and Spanish-Preferring Latino and Latina Individuals, 2018 to 2023, Among Patients Uninsured at Baseline

This supplemental material has been provided by the authors to give readers additional information about their work.

© 2026 Huguet N et al. *JAMA Network Open*.

eTable 1. Diagnostic, Lab LOINC, and *Current Procedural Terminology* Codes

**Colorectal Cancer**	
ICD-9/10 Codes	C18.0, C18.1, C18.2, C18.3, C18.4, C18.5, C18.6, C18.7, C18.8, C18.9, C19, C20, C49.A4, C49.A5, D01.0, D01.1, D01.2, Z85.030, Z85.038, Z85.040, Z85.048, 153, 153.1, 153.2, 153.3, 153.4, 153.5, 153.6, 153.7, 153.8, 153.9, 154, 154.1, 230.3, 230.4, V10.05, V10.06
**IBS/Crohn's**	
ICD-9/10 Codes	211.3, 531.90, 532.90, 534.90, 535.61, 537.9, 555, 555.0, 555.1, 555.2, 555.9, 556, 556.0, 556.1, 556.2, 556.3, 556.4, 556.5, 556.6, 556.8, 556.9, 558.9, 560.89, 560.9, 562.10, 564.1, 564.89, 564.9, 565.1, 569.3, 569.5, 569.81, 579.1, 713.1, K50.00, K50.011, K50.012, K50.013, K50.014, K50.018, K50.019, K50.10, K50.111, K50.112, K50.113, K50.114, K50.118, K50.119, K50.80, K50.811, K50.812, K50.813, K50.814, K50.818, K50.819, K50.90, K50.911, K50.912, K50.913, K50.914, K50.918, K50.919, K51.00, K51.011, K51.012, K51.013, K51.014, K51.018, K51.019, K51.20, K51.211, K51.212, K51.213, K51.214, K51.218, K51.219, K51.30, K51.311, K51.312, K51.313, K51.314, K51.318, K51.319, K51.412, K51.80, K51.811, K51.812, K51.813, K51.814, K51.818, K51.819, K51.90, K51.911, K51.912, K51.913, K51.914, K51.918, K51.919
**Indications for Diagnostic Screening**	
ICD-9/10 Codes	K63.5, R19.4, Z83.71, Z86.010, 211.3, 787.99, V12.72, V18.51, V76.51
**Colectomy**	
ICD-9/10 Codes	V45.72, Z90.49
CPT Codes	44140, 44141, 44143, 44144, 44145, 44146, 44147, 44150, 44151, 44152, 44153, 44155, 44156, 44157, 44158, 44160, 44204, 44205, 44206, 44207, 44210, 44211, 44212, 44213
Other EPIC codes	A44204, A44205, A44206, A44207, A44208, A44210, A44211, A44213, A45121, HX0030, SHX59, SUR275, SUR8
**Colonoscopy**	
ICD-9/10 Codes	Z12.11
CPT Codes	44388, 44389, 44390, 44391, 44392, 44393, 44394, 44397, 44401, 44402, 44403, 44404, 44405, 44406, 44407, 44408, 45355, 45378, 45379, 45380, 45381, 45382, 45383, 45384, 45385, 45386, 45387, 45388, 45389, 45390, 45391, 45392, 45393, 45398, C9779, G0105, G0121, G1029, G1031, G6019, G6020, G6024, G6025
Other EPIC codes	3604537801, 3604538001, 3604538101, 3604538401, 3604538501, 3604538801,7504537801, 7504537901, 7504538001, 7504538101, 7504538501, 7504539301, 7504539801, 848008, 9640081201, 9834537801, 9834538001, 9834538101, 9834538401, 9834538501, 9834538801, 9834539801, A44388, A44389, A44390, A44391, A44392, A44393, A44394, A44397, A44401, A44402, A44403, A44404, A44405, A44406, A44407, A44408, A45300, A45355, A45378, A45379, A45380, A45382, A45383, A45384, A45385, A45386, A45387, A45388, A45389, A45390, A45391, A45392, A45393, A45398, GI50, GI6, HX0060, LDC4520, LDCA1487, O34693, O34842, O55407, O56065, O56066, O60152, O65432, O65433, O65434, SHX174, TI2543, TI2544
Lab LOINC Codes	18746-8, 67166-9, 97100-2
**Colonography**	
CPT Codes	0066T, 0067T, 74263

© 2026 Huguet N et al. *JAMA Network Open*.

Other EPIC codes	A74261, A74263,3527426101, 3527426201, 3527426202, 3527426301, IMG1233, IMG1662, IMG2498, IMG2499, TI4096
Lab LOINC Codes	60515-4, 72531-7, 79069-1, 79071-7, 79101-2, 82688-3
**Sigmoidoscopy**	
CPT Codes	45330, 45331, 45332, 45333, 45334, 45335, 45337, 45338, 45339, 45340, 45341, 45342, 45345, 45346, 45347, 45349, 45350, G0104, G1030, G6022, G6023
Other EPIC codes	7504530701, 7504533201, 7504533501, A45303, A45305, A45307, A45308, A45309, A45315, A45320, A45321, A45327, A45330, A45331, A45332, A45333, A45334, A45335, A45337, A45338, A45339, A45340, A45341, A45342, A45345, A45346, A45347, A45350, GI14, GI5, SHX1649
Lab LOINC Codes	18753-4, 67166-9, 97101-0, 28027-1, 19796-2, LA15379-3
**FIT/FOBT**	
CPT Codes	82274
Other EPIC codes	LAS173, LBS002, LCS158, LCS359, LCS692, LDC1127, LDC1491, LDC3080, LDC3250, LDC4320, LES190, LR1211, LR2153, LV2180, LV3509, LV4773, LV5046, LV5391, LV5448, LV5578, LV5938, LV6535
Lab LOINC Codes	12504-7, 14564-9, 14565-6, 27396-1, 27401-9, 27925-7, 27926-5 , 56491-4, 57905-2, 58453-2, 80372-6, 12503-9, 14563-1, 2335-8, 29771-3, 56490-6, 77353-1, 77354-9

© 2026 Huguet N et al. *JAMA Network Open*.

eFigure 1. Event Study Plots to Test for Preperiod Parallel Trends

Panel A. English-preferring Latina and Latinos Colorectal Cancer Up-to-date Rates, Uninsured at baseline

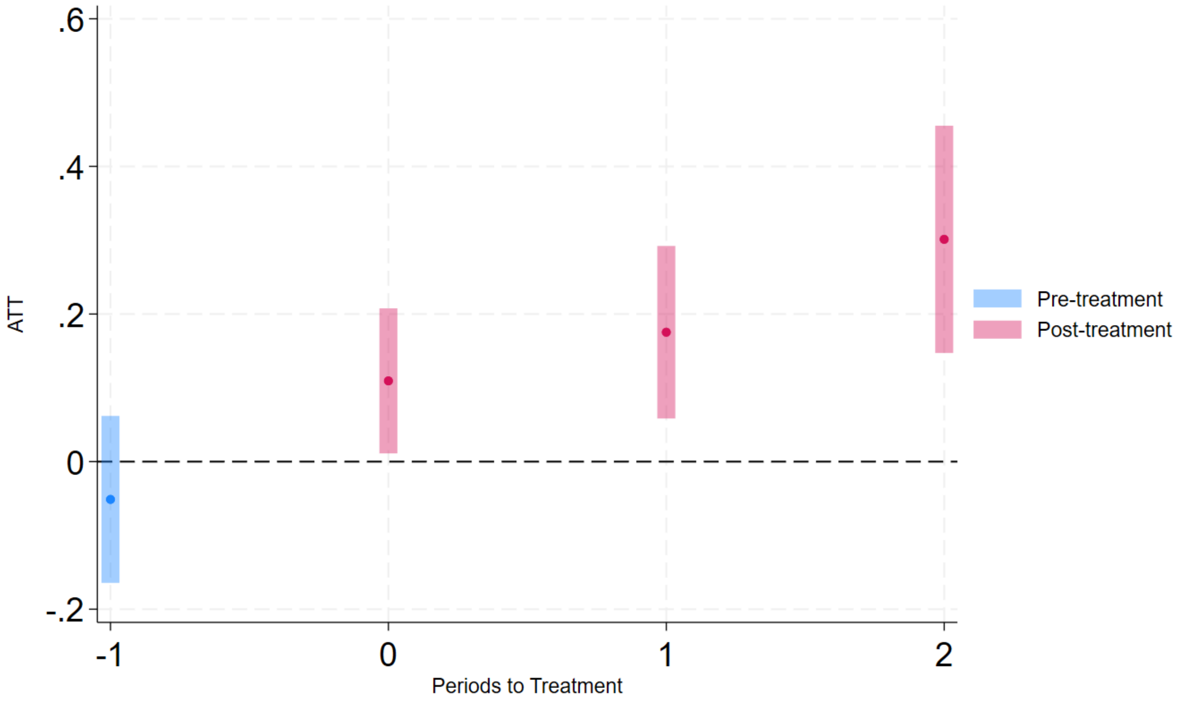

Panel B. Spanish-preferring Latina and Latinos Colorectal Cancer Up-to-date Rates, Uninsured at baseline

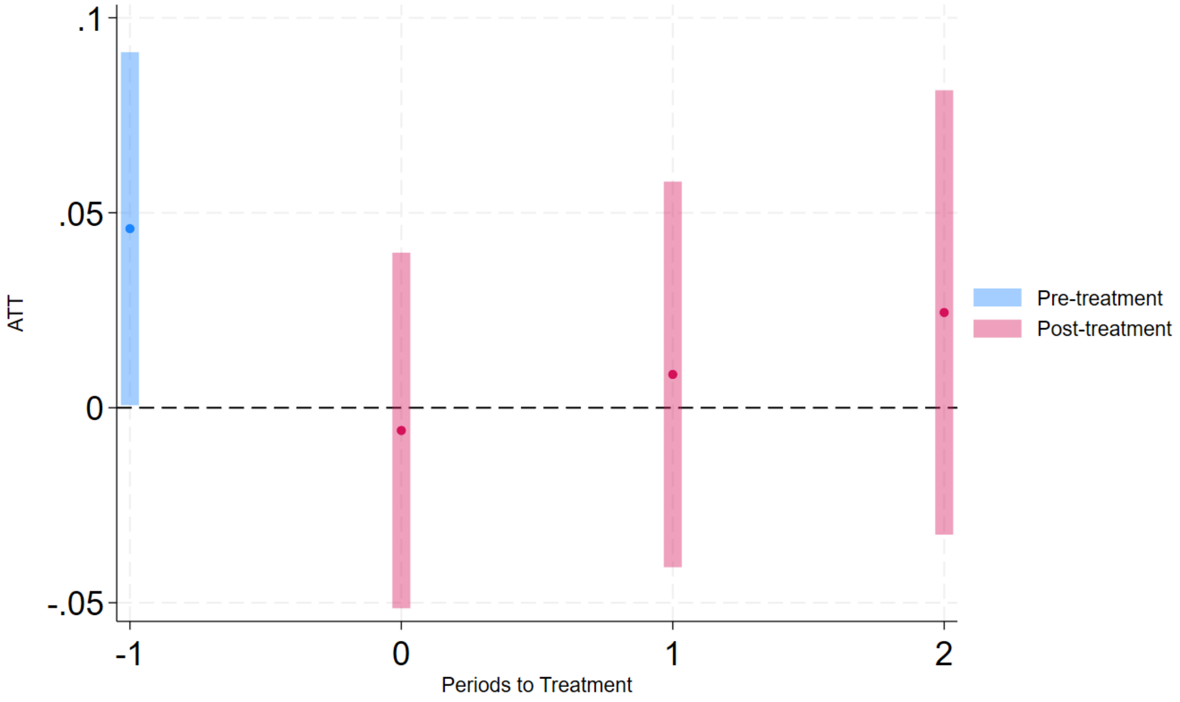

© 2026 Huguet N et al. *JAMA Network Open*.

Panel C. Spanish-preferring Latina Colorectal Cancer Up-to-date Rates, Uninsured at baseline

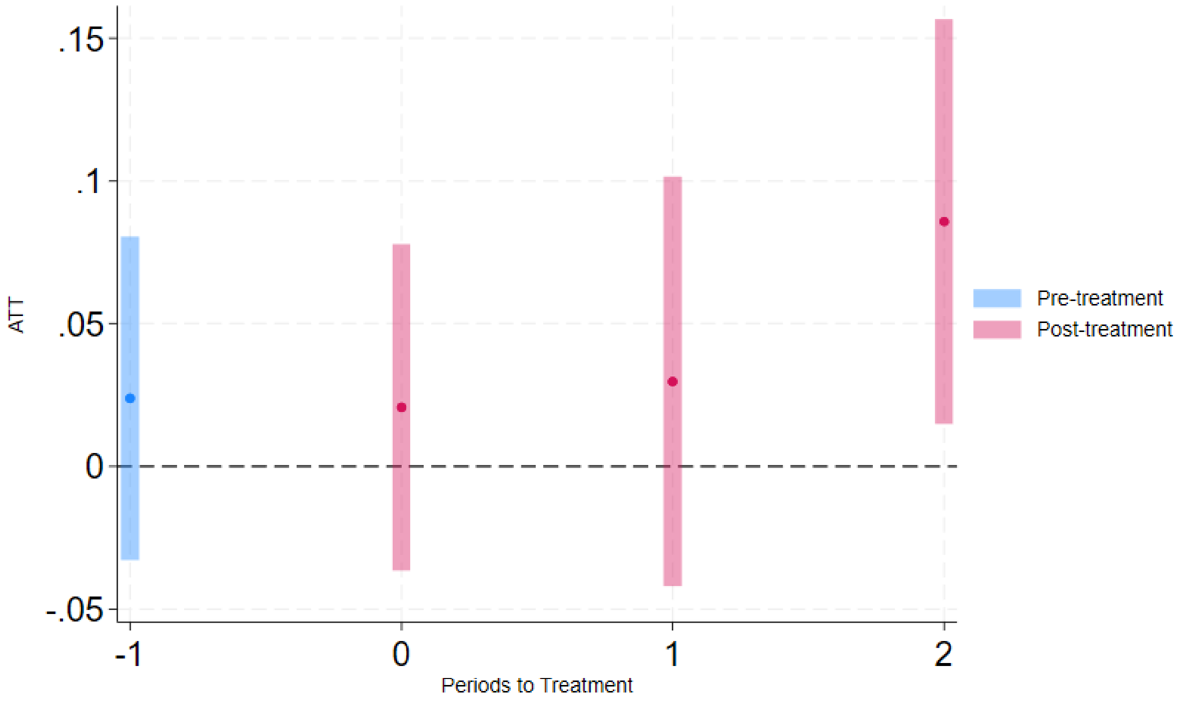

Panel D. English-preferring Latina Colorectal Cancer Up-to-date Rates, Uninsured at baseline

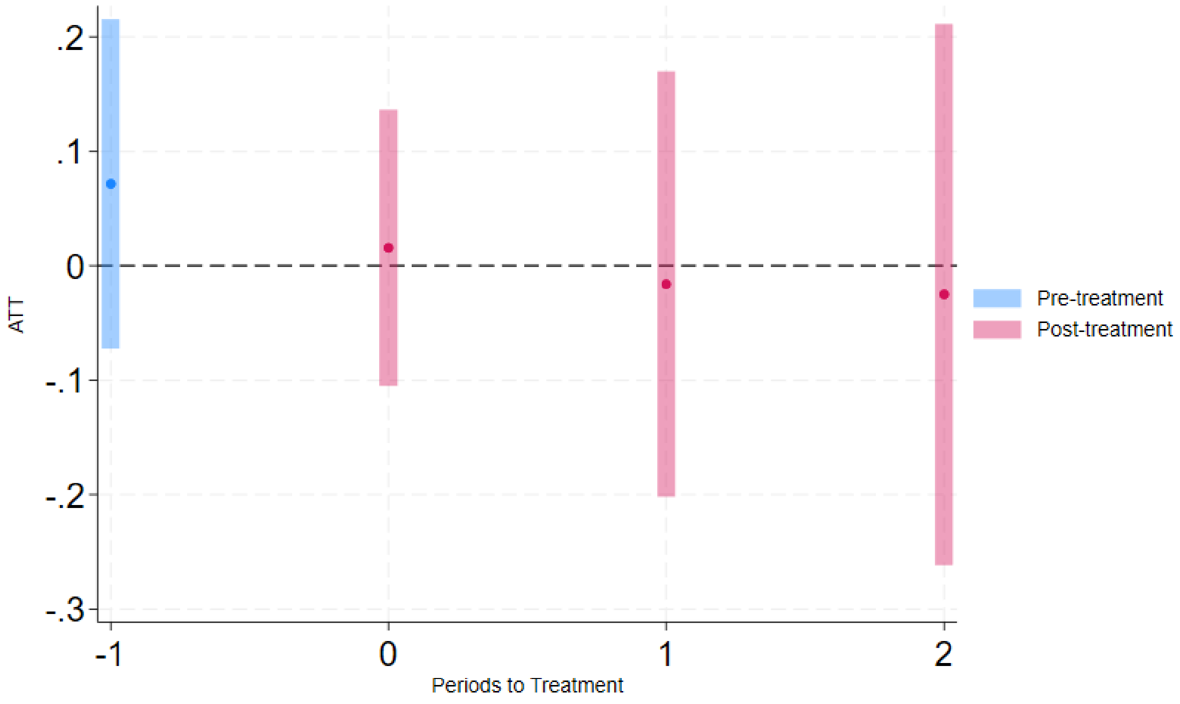

© 2026 Huguet N et al. *JAMA Network Open*.

Panel E. Spanish-preferring Latino Colorectal Cancer Up-to-date Rates, Uninsured at baseline

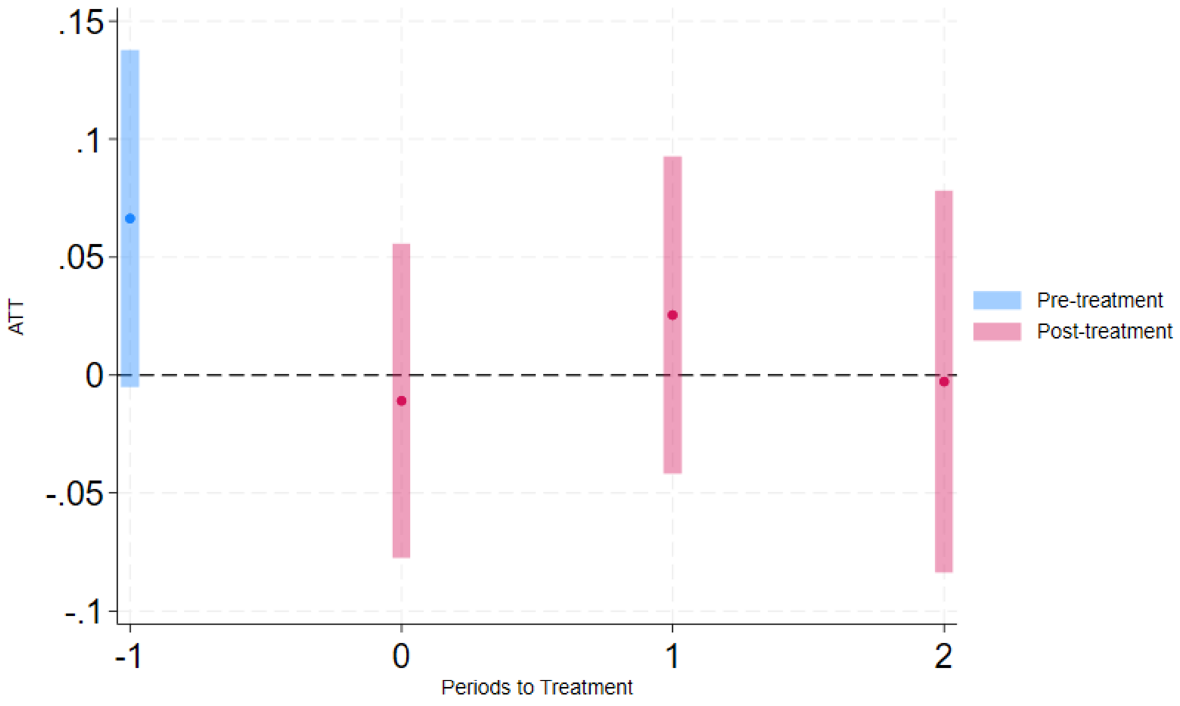

Panel F. English-preferring Latino Colorectal Cancer Up-to-date Rates, Uninsured at baseline

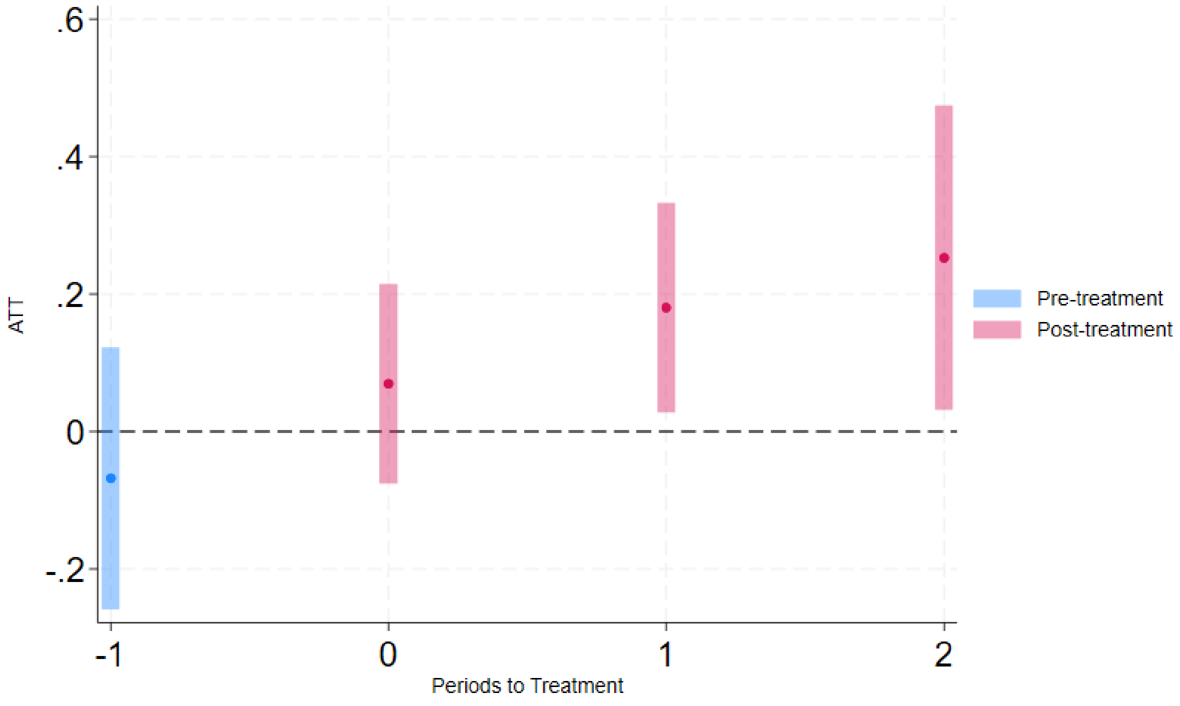

© 2026 Huguet N et al. *JAMA Network Open*.

Panel G. All Latino/as regardless of language Colorectal Cancer Up-to-date Rates, Uninsured at baseline, within California and Oregon

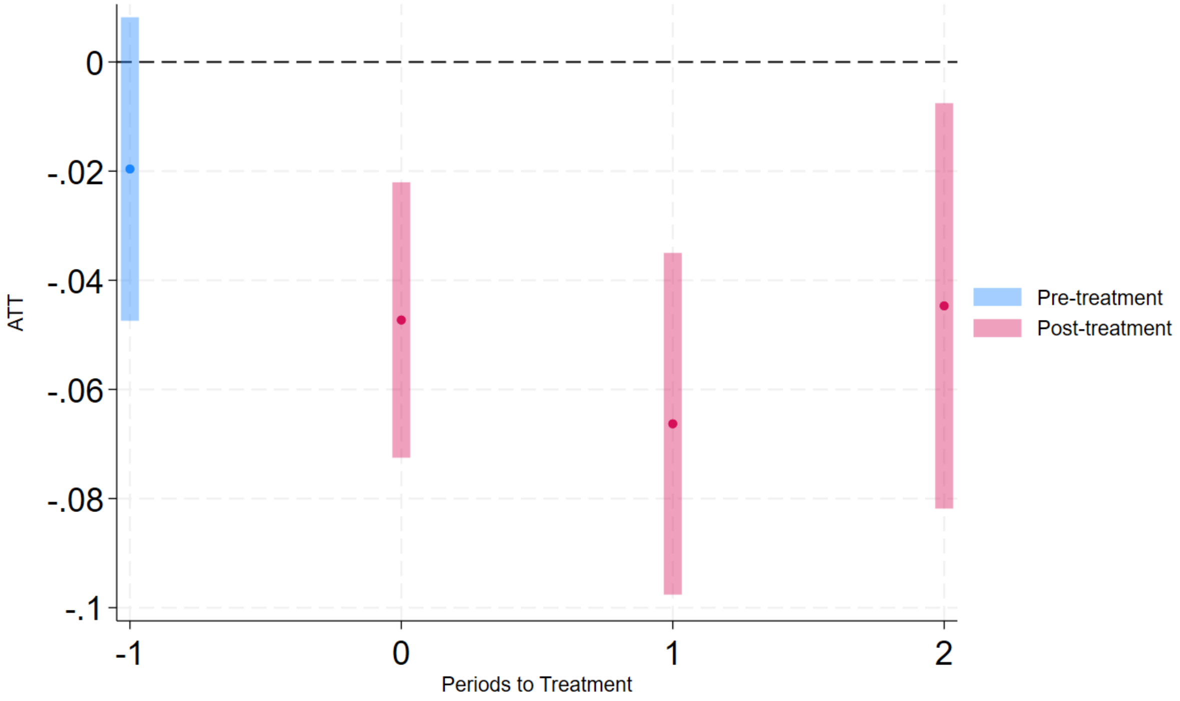

Panel H. English-preferring Latino/as Colorectal Cancer Up-to-date Rates, Uninsured at baseline, within California and Oregon

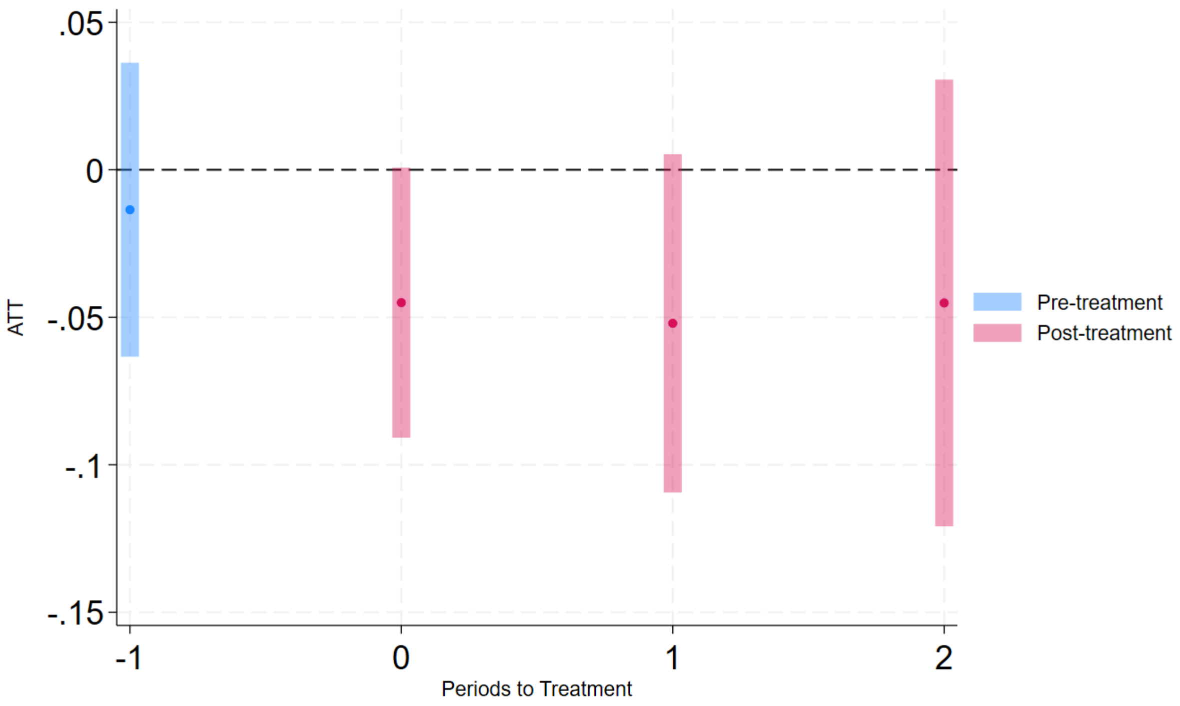

© 2026 Huguet N et al. *JAMA Network Open*.

Panel I. Spanish-preferring Latino/as Colorectal Cancer Up-to-date Rates, Uninsured at baseline, within California and Oregon

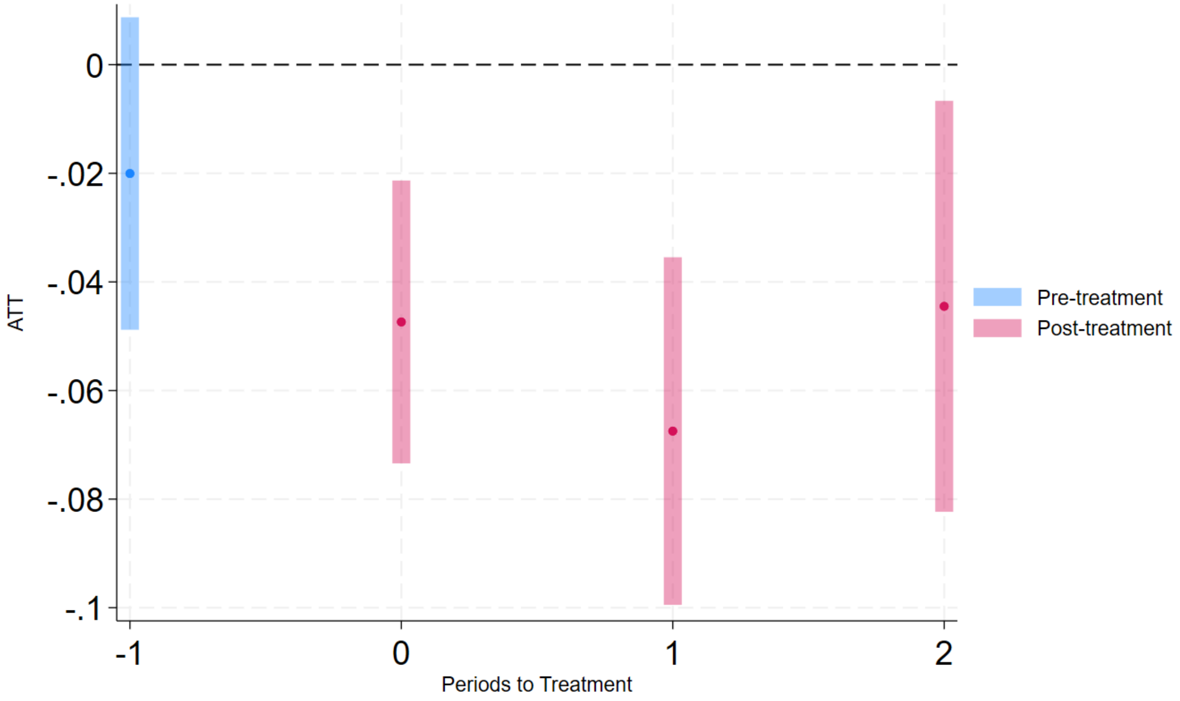

eTable 2. Statistical Testing of Preperiod Parallel Trends Assumptions

	**Group, p-value**		
	**Latino/a**	**Latina**	**Latino**
English-preferring, Amendment vs non-amendment states	0.3753	0.3288	0.4816
Spanish-preferring, Amendment vs non-amendment states	0.0468	0.4104	0.0684
	**Sensitivity analysis, Group, p-value[a]**		
	**All Latino/a**	**English-preferring Latino/as**	**Spanish-preferring Latino/as**
Non-Hispanic white vs Latino/as	0.1668	0.1721	0.5942

Note: P-values of the chi-squared statistics for the null hypothesis that average treatment effect on the treated estimations are statistically equal to zero during the 12 months prior to treatment. [a]Within California and Oregon

© 2026 Huguet N et al. *JAMA Network Open*.

eFigure 2. Unadjusted Percentage of Latino and Latina Individuals Up to Date on Colorectal Cancer Screening by Year and Sex Within the OCHIN Community Health Center Network

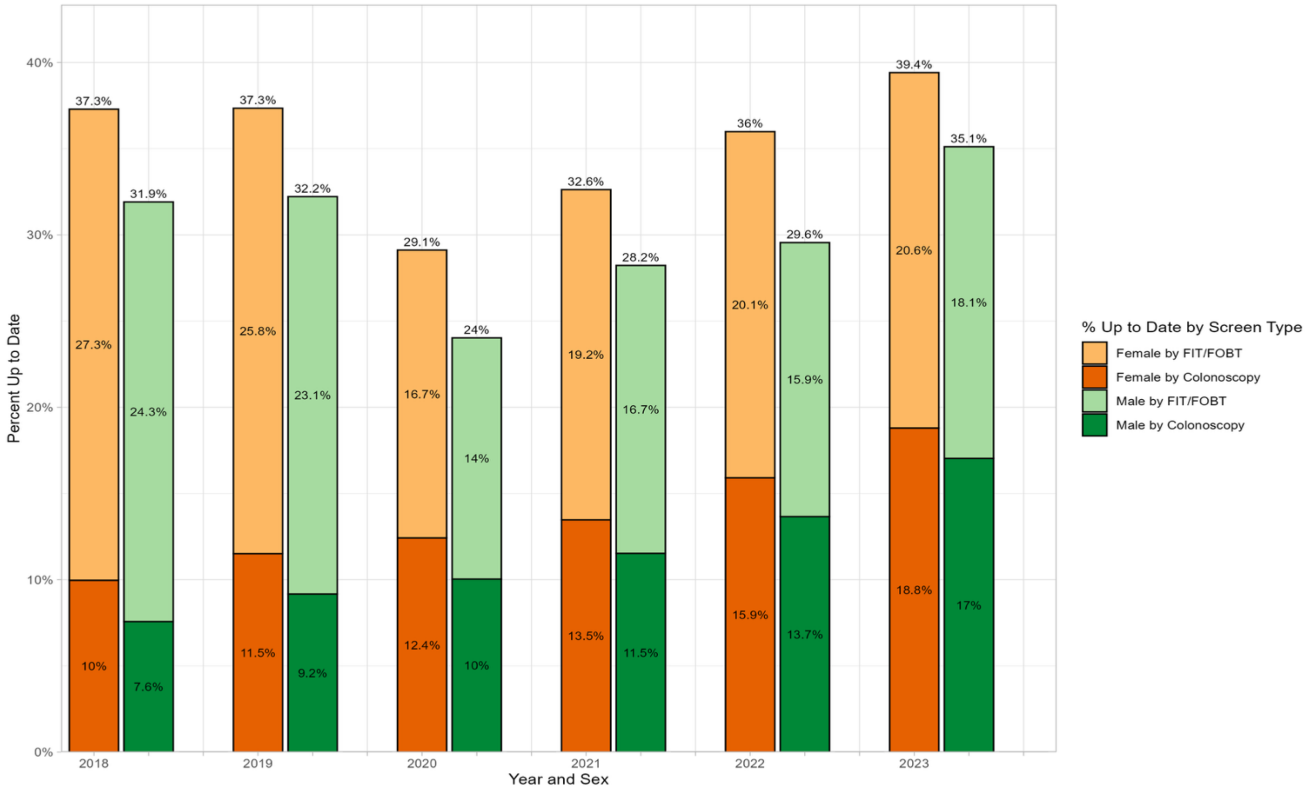

Latino/as were uninsured in the pre-period.
Bar-fill color depicts percentage up to date by screening method.

© 2026 Huguet N et al. *JAMA Network Open*.

eTable 3. Characteristics of English-Preferring Non-Hispanic White Individuals and English-Preferring and Spanish-Preferring Latino and Latina Individuals in California and Oregon Receiving Care in Community Health Clinics, 2018 to 2023

**Patient Characteristics**	**Total**<br>N=8,027	**Non-Hispanic White**<br>N=2,364	**Latino/a Prefers English**<br>N=419	**Latino/a Prefers Spanish**<br>N=5,244
Sex				
Female	4249 (52.9%)	1096 (46.4%)	171 (40.8%)	2982 (56.9%)
Male	3778 (47.1%)	1268 (53.6%)	248 (59.2%)	2262 (43.1%)
Age at Baseline Year				
50-54	4346 (54.1%)	1041 (44.0%)	228 (54.4%)	3077 (58.7%)
55-63	3681 (45.9%)	1323 (56.0%)	191 (45.6%)	2167 (41.3%)
Last Known Income				
≤138% FPL	4705 (58.6%)	1030 (43.6%)	224 (53.5%)	3451 (65.8%)
>138% FPL	2286 (28.5%)	896 (37.9%)	116 (27.7%)	1274 (24.3%)
No Information	1036 (12.9%)	438 (18.5%)	79 (18.9%)	519 (9.9%)
Homeless Ever During Study	555 (6.9%)	169 (7.1%)	37 (8.8%)	349 (6.7%)
Visits Per Year				
<2	4531 (56.4%)	1647 (69.7%)	252 (60.1%)	2632 (50.2%)
2-3	2341 (29.2%)	500 (21.2%)	110 (26.3%)	1731 (33.0%)
≥4	1155 (14.4%)	217 (9.2%)	57 (13.6%)	881 (16.8%)
Family History Colorectal Cancer	148 (1.8%)	70 (3.0%)	8 (1.9%)	70 (1.3%)
IBS or IBD Diagnosis	382 (4.8%)	127 (5.4%)	19 (4.5%)	236 (4.5%)
Chronic Conditions				
None	2605 (32.5%)	859 (36.3%)	132 (31.5%)	1614 (30.8%)
1	2847 (35.5%)	660 (27.9%)	151 (36.0%)	2036 (38.8%)
≥2	2575 (32.1%)	845 (35.7%)	136 (32.5%)	1594 (30.4%)
Colorectal Cancer Screening				
Up to date in 2019	2546 (31.7%)	629 (26.6%)	122 (29.1%)	1795 (34.2%)

FPL, federal poverty level; IBS, irritable bowel syndrome; IBD, inflammatory bowel disease
Data from 211 clinics in Amendment States = CA, OR. Pre-amendment (2018, 2019) and post-amendment (2021, 2022, 2023). Patients were uninsured at baseline.

© 2026 Huguet N et al. *JAMA Network Open*.

eFigure 3. Yearly Unadjusted Prevalence of Up-to-Date Colorectal Cancer Screening Among English-Preferring Non-Hispanic White Individuals and English-Preferring and Spanish-Preferring Latino and Latina Individuals, 2018 to 2023, Among Patients Uninsured at Baseline

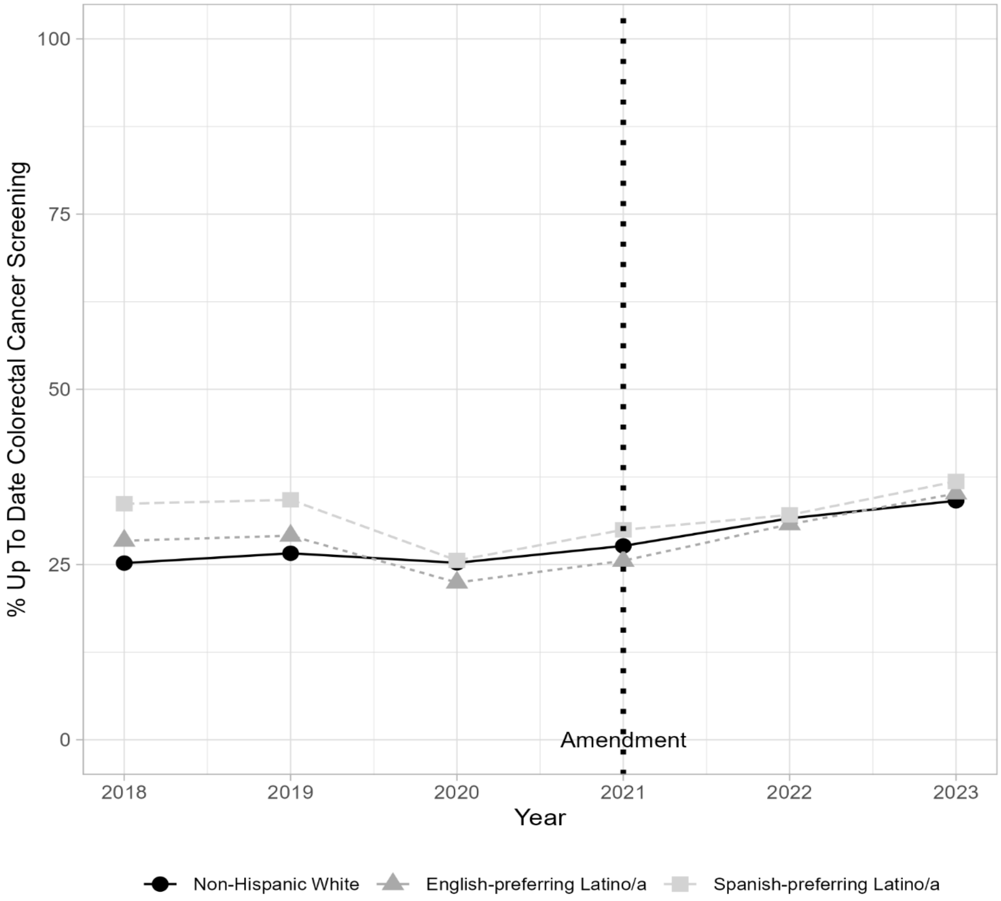

© 2026 Huguet N et al. *JAMA Network Open*.
