# Supplementary material for: Prevalence of Colorectal Cancer Screening Among Latino Adults Following the Medicaid Eligibility Amendment Expansion
Source: JAMA Netw Open. 2026 Feb 11;9(2):e2559100. doi: 10.1001/jamanetworkopen.2025.59100 (PMC12895285; doi:10.1001/jamanetworkopen.2025.59100)
Supplement: Supplement 2. — Data Sharing Statement [file jamanetwopen-e2559100-s002.pdf]

Data Sharing Statement

Huguet. Prevalence of Colorectal Cancer Screening Among Latino Adults Following the Medicaid Eligibility Amendment Expansion. *JAMA Netw Open*. Published February 11, 2026. doi:10.1001/jamanetworkopen.2025.59100

Data
Data available: No

Additional Information
Explanation for why data not available: Raw data underlying this article were generated from multiple health systems across institutions; restrictions apply to the availability and re-release of data under organizational agreements.
